# Patient activation, self-efficacy and usage of complementary and alternative medicine in cancer patients

**DOI:** 10.1007/s12032-022-01796-8

**Published:** 2022-09-07

**Authors:** Jutta Hübner, Saskia Welter, Gianluca Ciarlo, Lukas Käsmann, Emadaldin Ahmadi, Christian Keinki

**Affiliations:** 1 Medizinische Klinik II, Hämatologie und Internistische Onkologie, Universitätsklinikum Jena, Am Klinikum 1, 07747 Jena, Germany; 2 Department of Radiation Oncology, University Hospital, LMU Munich, Marchioninistrasse 15, 81377 Munich, Germany; 3 German Cancer Consortium (DKTK), Partner Site Munich, Munich, Germany

**Keywords:** Complementary and alternative medicine (CAM), Neoplasm, Patient activation, Self-efficacy, Communication, Patient–physician relationship

## Abstract

Complementary and alternative medicine (CAM) is used by many cancer patients by themselves. Therefore, we conducted a survey regarding the association between CAM, self-efficacy, and patient activation in adult cancer patients. A standardized questionnaire, consisted of the ASKU, the PAM 13-D, and a structured questionnaire on CAM usage from our own working group, was distributed to 880 potential participants. Six hundred and thirty-nine (639) patients (male 32.9%, female 63.2%; gynecological cancer 41%, gastrointestinal 19.2%, urogenital 15.6%) took part. 60% of all patients used CAM in the last 3 months (biological 73%, holistic 63%, mind–body methods 62%). Higher self-efficacy was associated with higher interest in CAM (*p* = 0.03), but not usage of CAM, compared to patients with lower self-efficacy (*p* = 0.099). Higher patient activation was associated with higher interest in CAM (*p* = 0.004) and usage of CAM (*p* = 0.012). Patients with higher activation significantly more often used homeopathy (*p* = 0.007), prayer (*p* = 0.002), yoga, etc. (*p* = 0.032), meditation (*p* = 0.002), low carb or ketogenic diets (*p* < 0.001) (but not vegan or other cancer diets). Higher patient activation is associated with higher usage of CAM. Focusing on patient activation as a goal in patient–physician relationship will help patients to adhere to a healthy lifestyle and to actively participate in the whole treatment process.

## Introduction

Complementary and alternative medicine (CAM) is often used by cancer patients [1, 2]. Goals with CAM are diverse. Patients want to strengthen themselves or do something for themselves. They aim at reducing side effects or to boost the immune system. Some use CAM to have better control of the cancer and/or not to leave out a chance [2, 3].

All in all, some CAM methods depend on the patient’s activity, while others are more dependent on a third party.

As CAM is most often initiated by the patient and at least a part of CAM methods depends on the patient’s action, a hypothesis is that CAM usage is a sign of patient activation and reinforces patient’s self-efficacy. If this is true, CAM might provide direct benefits by improving body or mental functions. Additionally, indirect benefits might be an increase in patient empowerment. Yet, as with some CAM methods the patient depends on the physician or expert as much as he/she does on the physician in case of a conventional treatment as radio- or chemotherapy, there are some doubts on this indirect effect at least for part of CAM.

With respect to personal characteristics of the patient and CAM usage, we have conducted several studies before. We did not find any association between the personal traits “Big Five” and CAM usage in general or categories of CAM [4]. In another study, patients using CAM often had a high external locus of control, while we did not find any association to a higher internal locus of control [4, 5]. We addressed the association of CAM and self-efficacy in two surveys as secondary endpoints and found no association [5].

To evaluate the association of CAM usage, self-efficacy, and patient activation in detail, we planned a nationwide survey on adult cancer patients in Germany.

## Methods

### Questionnaire

The questionnaire was composed of three validated questionnaires: the ASKU (Allgemeine Selbstwirksamkeit Kurzskala) [6], the PAM 13-D (Patient Activation Measure) [7], and the AKKOM (questionnaire on complementary and alternative medicine, developed by the working group Prevention and Integrative Oncology of the German Cancer Society) [2]. The development and testing of the questionnaire has been reported before [8]. The questionnaire was divided into seven sections containing 71 questions in total:

1. Demographic data (age, gender, education, marital status, religion, number of children, type and time of cancer diagnosis, cancer treatment and lay-etiologic concepts)
2. General self-efficacy scale
3. Patient Activation Measure
4. Complementary medicine (interest in, aims with and usage of complementary medicine sources of information)
5. Importance and satisfaction with information the patients got about their disease.

The questionnaire included different types of questions, such as closed, multiple choice as well as 4-point and 5-point Likert scales questions.

The anonymous questionnaire was distributed as print version.

### Patients

We included adult patients (≥ 18 years) with cancer attending an oncological outpatient clinic for the pilot study and patients attending a series of lectures on complementary medicine in different regions of Germany.

### Statistical analysis

Exporting data was managed using Excel 2019. We utilized IBM SPSS Statistics 27 for data collection and analysis. To analyze associations between variables, chi-square tests were used and *p* values smaller than 0.05 were considered significant.

### Ethics vote

Participation was voluntary and had no influence on the counseling on CAM or treatment. Written informed consent was given by filling in the questionnaire. The survey was accepted by an ethics committee.

## Results

### Demographic data

In total, 880 patients were addressed and 639 patients filled in the questionnaire (responsive rate: 72.6%, see Table 1). A third of the participants were male (32.9%) and two-thirds were female (63.2%). The leading cancer types were breast and gynecological cancer (41%), followed by gastrointestinal (19.2%) and urogenital cancer (15.6%).

**Table 1:** Demographic data (*N* = 639)

Category	Answer	*n* (%)
Gender	Male	210 (32.9)
	Female	404 (63.2)
	No answer	25 (3.9)
Age	< 30 years	4 (0.6)
	31–50 years	79 (12.4)
	51–70 years	388 (60.7)
	71–80 years	127 (19.9)
	> 80 years	39 (6.1)
	No answer	2 (0.3)
Education	Secondary school qualification	366 (57.3)
	High school graduation	48 (7.5)
	University diploma	184 (28.8)
	No answer	41 (6.4)
Type of cancer	Breast and gynaecological cancer	262 (41)
	Gastrointestinal cancer	123 (19.2)
	Urogenital cancer	100 (15.6)
	Others (head-neck, skin, brain, thyroid)	62 (9.7)
	Haemato-oncological cancer	40 (6.3)
	Lung cancer	36 (5.6)
	No answer	16 (2.5)

### CAM usage and goals with CAM

Nearly two-thirds of the patients reported already using CAM (294 of 484; 60.7%). The majority used CAM in order to strengthen the forces of their body (82.3%) or the immune system (78%). Improvement of well-being (58.7%) and detoxification (27.4%) were further reasons. Less than a fifth used CAM for healing cancer (18.4%). In contrast, reducing side effects, coping with mental stress, or supporting conventional medicine were only a goal for 6.1% of the CAM users. Detailed data are reported in Ciarlo et al. [8].

**Fig. 1:**
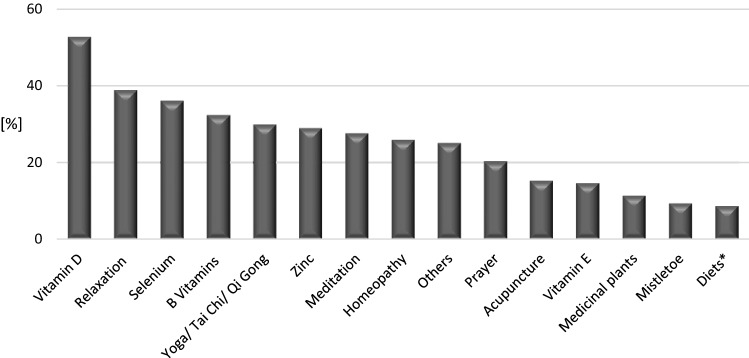
Usage of CAM (*N* = 355; multiple answers were possible), *low carb, ketogenic, vegan, Budwig, Breuss

The categories of CAM methods that were most often used included biologically based (73.2%), whole medical system (63.1%), and mind–body methods (62%) (see Fig. 1).

### Self-efficacy and patient activation measure

Overall, 601 (98.0%) participants answered all items of the self-efficacy scale (*M* = 3.99; SD = 0.752) and 627 (97.8%) answered all items of the patient activation measure (*M* = 67.91; SD = 18.27). Detailed data are reported in Welter et al. [9].

Higher self-efficacy was associated with higher interest in CAM (*p* = 0.03). Yet, patients with higher self-efficacy do not use CAM more often than those with lower self-efficacy (*p* = 0.099). Some goals with CAM are associated with a higher self-efficacy. These goals are strengthening one’s own forces (*p* = 0.035), healing of the cancer (*p* = 0.008), or to do something for oneself (*p* = 0.039). Considering the methods used, patients with higher self-efficacy more often use homeopathy (*p* = 0.035), prayer (*p* = 0.040), or relaxation (*p* = 0.018). Yet, there was no association to meditation, yoga, or biologically based methods with the exception of vegan diet (*p* = 0.030). Also for cancer diets (low carb, ketogenic, Budwig or Breuss), there was no association.

Higher patient activation was associated with higher interest in CAM (*p* = 0.004) and usage of CAM (*p* = 0.012). Considering the different goals, no association was found to patient activation. With respect to CAM methods, patients with higher activation significantly more often reported using homeopathy (*p* = 0.007), prayer (*p* = 0.002), Yoga (*p* = 0.032), meditation (*p* = 0.002), low carb or ketogenic diets (*p* < 0.001) (but not vegan or other cancer diets).

## Discussion

CAM usage is high in cancer patients and the type of CAM used was similar to former surveys with a preponderance of biologically based methods, mostly micronutrients [9]. Main goals for using CAM were strengthening the forces of the own body or the immune system. These goals are similar to former surveys [2, 3] even on an international level [10–12]. These goals may be categorized as general goals in contrast to specific ones as reducing side effects. This was also reported by Wode and colleagues [12]. In line with this, two studies report a higher self-efficacy in patients in mind–body intervention groups [13, 14]. Moreover, patients turn to mind–body methods which often aim at improving well-being [15].

In contrast, reduction of side effects was named only by a small minority. Also Koenig et al. reported an improvement of tolerability to be a less important goal [16]. Accordingly, we have a contrast between patients’ needs and the endpoints in most clinical studies on biological-based CAM which aim at specific side effects.

Another important point in this context is that higher self-efficacy goes along with a higher effect of alternative treatments as Reiki which depend on a high placebo effect [17]. In fact, this would also explain the affinity of high self-efficacious patients to homeopathy.

Considering patient activation, a higher activation is associated with higher interest in CAM and usage of CAM. We found a broad range of methods being associated with higher activation which are quite similar to the methods used by patients with higher self-efficacy. Yet, considering the different goals, no association was found to patient activation. Loquai et al. have shown that patients using CAM are more likely to be physically active and to look for psychosocial support or contact to self-help [18]. Accordingly, Hibbard et al. reported higher activation in patients better coping with side effects [19].

All in all, a strategy to discuss CAM with cancer patients may be to start with agreeing on the goals before discussing special CAM methods. Moreover, patients attitude and needs for holistic (mind, body, soul) care should be addressed. Considering these goals and attitudes, patients should be referred to the best matching methods for which respective endpoints have been studied.

## Limitation

The most important limitation of our survey is the recruitment of the patients as we addressed participants of lectures on CAM. Thus, interest in CAM has to be expected to be high. Yet, the rate of CAM users is nearly in line with recent German data [1, 3, 20].

## Conclusion

Physicians might focus on patient activation as a goal in patient–physician relationship which will help patients to adhere to a healthy lifestyle and to participate in the whole treatment process.

Also, self-efficacy may entail positive effects if it is directed on goals such as coping with cancer and distress which may improve quality of life [17, 21, 22]. Providing information on mind–body techniques or self-help group participation may focus these patients on beneficial activities [23].

## Data Availability

The datasets generated and analyzed during the current study are available from the corresponding author on reasonable request.
